# Impact of Hiv-Associated Conditions on Mortality in People Commencing Anti-Retroviral Therapy in Resource Limited Settings

**DOI:** 10.1371/journal.pone.0068445

**Published:** 2013-07-23

**Authors:** Catherine S. Marshall, Andrea J. Curtis, Tim Spelman, Daniel P. O’Brien, Jane Greig, Leslie Shanks, Philipp du Cros, Esther C. Casas, Marcio Silveira da Fonseca, Eugene Athan, Julian H. Elliott

**Affiliations:** 1 Infectious Diseases Unit, Alfred Hospital, Melbourne, Australia; 2 Department of Epidemiology and Preventive Medicine, Monash University, Melbourne, Australia; 3 Centre for Population Health, Burnet Institute, Melbourne, Australia; 4 Public Health Department, Médecins Sans Frontières, Amsterdam, Netherlands; 5 Department of Infectious Diseases, Geelong Hospital, Geelong, Australia; 6 Department of Medicine and Infectious Diseases, Royal Melbourne Hospital, University of Melbourne, Melbourne, Australia; 7 Manson Unit, Médecins Sans Frontières, London, United Kingdom; 8 School of Medicine, Deakin University, Geelong, Australia; Temple University School of Medicine, United States of America

## Abstract

**Objectives:**

To identify associations between specific WHO stage 3 and 4 conditions diagnosed after ART initiation and all cause mortality for patients in resource-limited settings (RLS).

**Design, Setting:**

Analysis of routine program data collected prospectively from 25 programs in eight countries between 2002 and 2010.

**Subjects, Participants:**

36,664 study participants with median ART follow-up of 1.26 years (IQR 0.55–2.27).

**Outcome Measures:**

Using a proportional hazards model we identified factors associated with mortality, including the occurrence of specific WHO clinical stage 3 and 4 conditions during the 6-months following ART initiation.

**Results:**

There were 2922 deaths during follow-up (8.0%). The crude mortality rate was 5.41 deaths per 100 person-years (95% CI: 5.21–5.61). The diagnosis of any WHO stage 3 or 4 condition during the first 6 months of ART was associated with increased mortality (HR: 2.21; 95% CI: 1.97–2.47). After adjustment for age, sex, region and pre-ART CD4 count, a diagnosis of extrapulmonary cryptococcosis (aHR: 3.54; 95% CI: 2.74–4.56), HIV wasting syndrome (aHR: 2.92; 95%CI: 2.21 -3.85), non-tuberculous mycobacterial infection (aHR: 2.43; 95% CI: 1.80–3.28) and *Pneumocystis* pneumonia (aHR: 2.17; 95% CI 1.80–3.28) were associated with the greatest increased mortality. Cerebral toxoplasmosis, pulmonary and extra-pulmonary tuberculosis, Kaposi’s sarcoma and oral and oesophageal candidiasis were associated with increased mortality, though at lower rates.

**Conclusions:**

A diagnosis of certain WHO stage 3 and 4 conditions is associated with an increased risk of mortality in those initiating ART in RLS. This information will assist initiatives to reduce excess mortality, including prioritization of resources for diagnostics, therapeutic interventions and research.

## Introduction

Despite increasing access to HIV treatment in resource-limited settings (RLS), patients commencing anti-retroviral therapy (ART) in these settings have been shown to have an increased risk of mortality in the first months of therapy compared with those in high-income countries [1], [2], although this difference in mortality risk reduces with time on ART [1].

A number of factors have been associated with mortality during early ART in RLS. These include WHO clinical stage, CD4 count, body weight, anemia, male sex and lack of free access to treatment [3]–[6]. Opportunistic infections have been recognized as important causes of early mortality in those initiating ART in RLS [7], [8], but limited data are available regarding the relative impact of specific HIV-associated conditions on mortality. Both prevalent and incident tuberculosis (TB) have been associated with a greater than two fold increased risk of mortality during ART in a South African cohort [9] and cryptococcal meningitis has a high mortality in RLS [10], [11].

Defining the relative impact of specific HIV associated conditions on mortality during early ART will assist efforts to reduce the excess mortality associated with this period of HIV care by assisting program planners in strategic decision making and prioritizing resources aimed at the prevention, diagnosis and treatment of these conditions both before and after the initiation of ART. The objective of this study was therefore to determine the association between specific WHO stage 3 and 4 conditions diagnosed after initiation of ART and all cause mortality on ART.

## Materials and Methods

### Patients and Procedures

We analyzed data prospectively collected between 2002 and 2010 from patients commencing ART in 25 Médecins Sans Frontières (MSF) supported treatment programs in Democratic Republic of Congo, Ethiopia, India, Ivory Coast, Moldova, Myanmar, Nigeria, Republic of Congo, Zambia and Zimbabwe. Eligible patients were not previously exposed to ART and aged 15 years and over. Eligibility criteria for ART and first-line regimens were based on WHO guidelines [12], [13] and were generally non-nucleoside reverse-transcriptase inhibitor based. All programs provided full blood examination, alanine transaminases and CD4 cell count prior to ART initiation. Clinical consultations and intensive adherence counseling sessions were provided prior to and during ART but adherence was not routinely recorded. Daily co-trimoxazole prophylaxis was given to patients with WHO clinical stage 2, 3 or 4 disease or those with CD4 cell count less than 350 cells/μL. TB prophylaxis was not given routinely. Mycobacterium avium complex (MAC) disease was actively screened for only in Myanmar where primary prophylaxis was also provided with azithromycin if CD4 count was <50 cells/μL. CD4 cell counts were monitored using automated methods but HIV viral load testing was not routinely performed. All care was free including ART, treatment and prophylaxis of opportunistic infections, clinical consultations and laboratory monitoring. Treatment sites included both hospital settings and peripheral health clinics. Patients were considered lost to follow-up (LFU) if they missed their next scheduled appointment by more than 2 months.

Clinical care was provided by medical doctors and clinical officers. Diagnosis of HIV-associated conditions was based on WHO guidelines [12] using basic laboratory investigations including biochemistry, haematology, sputum microscopy for acid fast bacilli and gram stain, chest x-ray and India ink examination of cerebrospinal fluid. Some sites had access to investigations such as cryptococcal antigen tests and abdominal ultrasound but these were not routinely available at all sites.

Treatment of opportunistic infections was based on standardized MSF guidelines [14] and modified depending on availability of appropriate drugs. Patients with cryptococcosis usually received treatment with amphotericin B, although fluconazole was used at lower than currently recommended doses (400 mg daily for induction) when amphotericin B was not available. 5-flucytosine was not available at any site. TB was treated according to WHO guidelines [15]. Drug resistant TB could not be diagnosed or treated at most sites. Sites did not have access to mycobacterial culture, but clinically suspected MAC infection was able to be treated with clarithromycin and ethambutol. Toxoplasmosis was usually treated with co-trimoxazole and in most sites bleomycin was available to treat Kaposi’s sarcoma. Patients with malnutrition received therapeutic nutritional support.

At the initial consultation, information about medical history and socio-demographic characteristics were collected. Clinical and therapeutic information, including HIV associated conditions, weight, and duration of follow up was recorded at subsequent consultations. All information was collected on standard forms and entered into a standardised electronic database. Due to a lack of pre-ART data in a number of sites, this data was not included in the analysis.

### Statistical analysis

All analyses were performed using de-identified data. Conditions newly diagnosed during the six months following ART initiation were included in the analysis. If patients were diagnosed with multiple HIV associated conditions they were all included, however each condition was only included once. We used a modified intention to treat analysis, ignoring treatment changes and interruptions. The primary outcome of the analysis was all-cause mortality and loss to follow-up (LFU) was a secondary outcome.

We used a Cox proportional hazards model to identify the factors associated with mortality during the follow up period. Covariates included age, sex, pre-ART CD4 cell count, geographical region, weight and the occurrence of specific individual WHO stage 3 and 4 conditions in the 6 months following ART initiation. The multivariate model included all factors that were associated with mortality on univariate analysis (*P*<0.05) and were associated with greater than 10 deaths. Incomplete ascertainment of height precluded the inclusion of body mass index in the analyses. A similar model was used to identify factors associated with being lost to follow-up, although the overall occurrence of WHO stage 3 and 4 conditions, rather than specific conditions, were examined as co-variates. Hazard proportionality was assessed through analysis of scaled Schoenfeld residuals. Interactions between model predictors were also tested.

In the setting of 7.3% LFU a competing risks extension of the Cox models were derived to assess the impact, if any, of LFU on the study’s ability to observe mortality.

We used Kaplan Meier survival analysis to compare survival of those with the diagnosis of a WHO stage 3 or 4 condition within 6 months of ART initiation and those without. Time was measured from the start of ART and was censored at date of death; date of last recorded visit or at the program end date. STATA version 11 (StataCorp, College Station, Texas, USA) was used for the analysis.

### Ethics Statement

The study was approved by the MSF ethics review board, the Alfred Hospital Human Research and Ethic Committee (Approval 331.09) and the Monash University Human Ethics Committee (Approval 2010000219). A waiver of the requirement for informed consent was granted by these review boards on the basis that the analysis is observational, retrospective, performed on anonymous data and will not impact on the care of patients.

## Results

### Baseline Characteristics

A total of 36,664 patients commenced ART for the first time and were followed for a median of 1.26 years (IQR: 0.55 to 2.27; Table 1). When compared to patients treated in Asia, African participants were more likely to be female (56.1% vs. 40.4%, *P*<0.001), have less advanced HIV disease, with a higher baseline CD4 count (150 vs. 102 cells/μL, *P*<0.001) and be less likely to have WHO stage 4 disease (10.9% vs. 32.8%, *P*<0.001).

**Table 1:** Baseline Characteristics of Participants.

	Overall	Africa	Asia
No. participants (%)	36,664(100)	22,951(62.5)	13,073(35.7)
Median Follow-up: Years (IQR)	1.26(0.55–2.27)	1.07(0.37–2.07)	1.56(0.87–2.51)
Median age (IQR)	33(28–39)	35(28–42)	32(28–38)
Female sex (%)	18,405(50.2)	12,877(56.1)	5,278(40.4)
Median Baseline CD4 cell count (IQR)^1^	108(39–198)	150(92–179)	102(33–191)
WHO stage: 1 (%)	7226(19.7)	5362(23.4)	1750(13.4)
2 (%)	5765(15.7)	4498(19.6)	1326(10.14)
3 (%)	15,310 (41.8)	9415(41.0)	5667(43.3)
4 (%)	7131(19.4)	2489(10.9)	4285(32.8)
stage unknown	1,232(3.4)	1,187(5.2)	45(0.3)

1 CD4 cell count available for 32,091 patients (87.5%).

### Mortality

**Figure 1:**
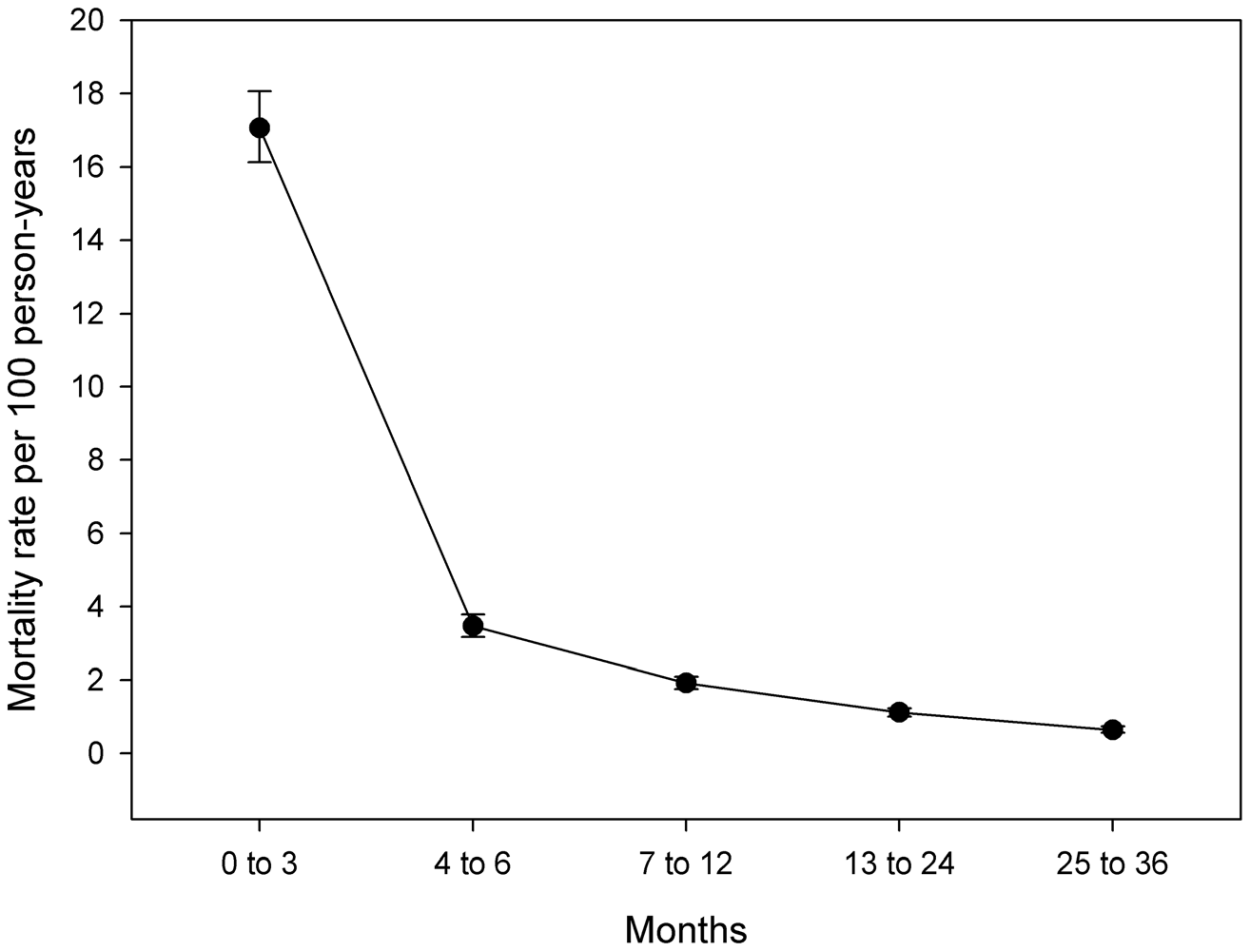
Mortality rates in time periods after commencement of anti-retroviral therapy.

There were a total of 2922 deaths during the follow-up period, giving an overall crude mortality rate of 5.41 deaths per 100 person-years. There were 1217(41.6%) deaths during the first 3 months following ART initiation, giving a mortality rate of 17.07 per 100 person years (95% CI: 16.13 to 18.06) that fell to 0.64 per 100 person years (95% CI: 0.56 to 0.74) in the period after 24 months of therapy (Figure 1). During the first 12 months on ART 6.1% (n = 2,241) of participants died.

### Predictors of Mortality

**Figure 2:**
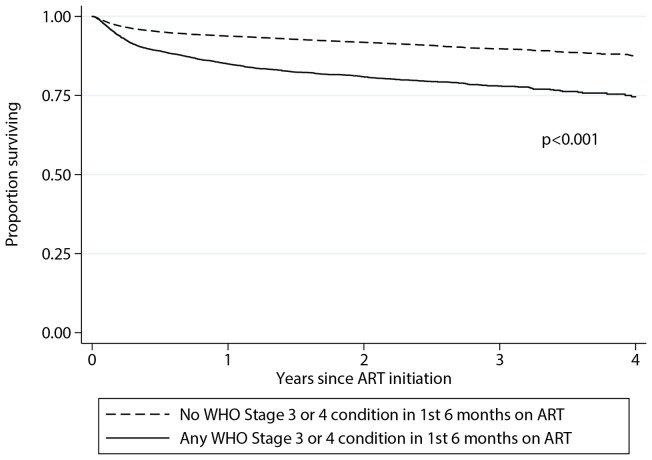
Kaplan-Meier survival analysis comparing those with a diagnosis of any WHO stage 3 or 4 condition within the first 6 months after ART initiation and those without.

A diagnosis of any WHO stage 3 or 4 condition during the first 6 months on ART was associated with greater than 2 fold increased risk of death on univariate analysis (HR: 2.21; 95% CI: 1.97 to 2.47; *P*<0.001). The Kaplan Meier survival curve (Figure 2) shows that the majority of the excess mortality occurred during the first six month period on ART.

General patient characteristics that were associated with mortality on both univariate and multivariate analysis included African region, male sex, older age and lower baseline CD4 cell count (Table 2). Weight <45kg was associated with increased mortality on univariate analysis (HR: 1.57; 95% CI: 1.42 to 1.73; *P*<0.001) but was not included in the multivariate model because it caused the model to violate hazard proportionality.

**Table 2:** Factors associated with mortality after initiation of ART.

	Number of Cases	Number of deaths	Unadjusted HR (95% CI)	P-value	Adjusted HR (95% CI)	P-value
Region: Africa	-	1821	1.22(1.13–1.32)	<0.001	1.30(1.20–1.41)	<0.001
Asia	-	1036	1.0		1.0	
Gender: Male	-	1542	1.61(1.5–1.73)	<0.001	1.55(1.43–1.67)	<0.001
Female	-	1380	1.0		1.0	
Age (per year)	-	-	1.03(1.02–1.04)	<0.001	1.02(1.02–1.03)	<0.001
CD4 cell count (per 100)	-	-	0.71(0.68–0.74)	<0.001	0.75(0.72–0.78)	<0.001
Weight: <45kg	-	1039	1.57(1.42–1.73)	<0.001	-	
45–49 kg	-	626	1.03(0.93–1.15)	0.547	-	
50–59kg	-	574	0.70(0.63–0.78)	<0.001	-	
≥60kg	-	678	1.0		-	
**WHO Stage 3 Conditions**						
Unexplained anemia/ neutropenia/thrombocytopenia	84	27	4.39(3.01–6.42)	<0.001	1.59(0.99–2.55)	0.054
Severe bacterial pneumonia	239	58	2.49(1.92–3.22)	<0.001	1.31(0.99–1.74)	0.06
Other Severe bacterial infections	183	38	2.45(1.78–3.38)	<0.001	1.26(0.88–1.80)	0.212
Pulmonary TB	1436	253	2.02(1.77–2.30)	<0.001	1.49(1.30–1.70)	<0.001
Unexplained prolonged fever	203	36	1.78(1.28–2.48)	0.001	0.83(0.57–1.22)	0.348
Oral Candidiasis	1448	247	1.73(1.52–1.98)	<0.001	1.19(1.03–1.36)	0.017
Unexplained chronic diarrhoea	396	64	1.61(1.25–2.06)	<0.001	1.07(0.82–1.39)	0.626
Weight loss >10% body weight	476	68	1.55(1.22–1.97)	<0.001	1.03(0.79–1.34)	0.819
Oral hairy leukoplakia	422	57	1.31(1.00–1.69)	0.048	1.04(0.79–1.38)	0.776
**WHO Stage 4 conditions**						
Cryptococcosis extra-pulmonary	179	72	5.19(4.11–6.55)	<0.001	3.54(2.74–4.56)	<0.001
HIV wasting syndrome	166	63	4.91(3.82–6.30)	<0.001	2.92(2.21–3.85)	<0.001
Pneumocystis jiroveci pneumonia	104	35	3.65(2.62–5.09)	<0.001	2.17(1.49–3.16)	<0.001
Kaposi’s sarcoma	84	28	3.62(2.49–5.25)	<0.001	1.84(1.19–2.84)	0.006
Toxoplasmosis of the brain	97	32	3.60(2.54–5.10)	<0.001	1.89(1.26–2.83)	0.002
Non TB mycobacteria infection	189	53	3.01(2.29–3.95)	<0.001	2.43(1.80–3.28)	<0.001
Candidiasis of the oesophagus	292	78	2.87(2.29–3.60)	<0.001	1.87(1.47–2.38)	<0.001
Penicilliosis Marneffei	74	19	2.64(1.68–4.15)	<0.001	1.16(0.65–2.06)	0.616
Extra-pulmonary TB	380	82	2.26(1.81–2.81)	<0.001	1.42(1.12–1.80)	0.004
Mucocutaneous Herpes Simplex	106	12	1.03(0.59–1.82)	0.909	-	

Of the specific WHO stage 3 and 4 conditions, extra-pulmonary cryptococcosis was associated with the greatest increased risk of death on multivariate analysis (aHR: 3.54; 95% CI: 2.74 to 4.56), followed by HIV wasting syndrome (aHR: 2.92; 95% CI: 2.21 to 3.85). The other stage 4 conditions significantly associated with mortality on multivariate analysis were non-tuberculous mycobacterial infection, *Pneumocystis* pneumonia, cerebral toxoplasmosis, oesophageal candidiasis, Kaposi’s sarcoma and extra-pulmonary TB. These conditions were all associated with an approximately 2 fold increased risk of death (aHR: 1.83 to 2.43) except for extra-pulmonary TB which was associated with a smaller increase in risk of death (aHR: 1.42; 95% CI: 1.12 to 1.8). Penicilliosis was associated with increased mortality on univariate analysis but not on multivariate analysis and chronic mucocutaneous herpes simplex infection was not associated with an increased risk of death in this analysis.

WHO stage 3 conditions were less strongly associated with mortality than the stage 4 conditions (unadjusted HR: 1.88; 95% CI: 1.65 to 2.14 vs. HR: 3.59; 95% CI: 2.97 to 4.33). Unexplained anemia, neutropenia or thrombocytopenia was most strongly associated with mortality on univariate analysis (HR: 4.39; 95% CI: 3.01 to 6.42; *P*<0.001), although the association was weaker in the multivariate analysis (aHR: 1.59; 95% CI: 0.99 to 2.55; *P* = 0.054). Pulmonary TB, severe bacterial pneumonia and other severe bacterial infections were also strongly associated with mortality in univariate analyses and were associated with aHR of 1.26 to 1.49 in multivariate analyses, although only the result for pulmonary TB was statistically significant. Oral candidiasis (aHR: 1.19; 95% CI: 1.03 to 1.36; *P*  = 0.017) was the only other WHO stage 3 condition that remained significantly associated with mortality on multivariate analysis.

The results of the competing risks analysis, performed to assess the impact of LFU on the mortality analysis, were largely consistent with the primary analysis (data not shown). There were no differences in the conditions associated with mortality on univariate analysis. In the multivariate analysis oral candidiasis was no longer associated with an increased mortality (aHR: 1.12; 95% CI: 0.94–1.34; *P* = 0.215) but pyomyositis (aHR: 1.67; 95% CI: 1.01 to 2.78; *P* = 0.049) and *Penicilliosis Marneffei* (aHR: 1.73; 95% CI: 1.03 to 2.88; *P* = 0.037) both became significantly associated with mortality.

### Loss to follow-up

A total of 2672(7.3%) patients were LFU, giving an overall LFU rate of 4.96 per 100 person-years (95% CI: 4.77 to 5.15). A diagnosis of any WHO stage 4 condition, African region and lower baseline CD4 cell were associated with increased risk of LFU on both univariate and multivariate analysis (Table 3). Female sex and a diagnosis of any WHO stage 3 condition were associated with increased risk of LFU on univariate analysis but these did not remain significant on multivariate analysis.

**Table 3:** Predictors of loss to follow-up after ART initiation.

	Unadjusted HR (95% CI)	P-value	Adjusted HR (95% CI)	P-value
Region: Asia	1.00		1.00	
Africa	3.09(2.80–3.40)	<0.001	2.83(2.37–3.39)	<0.001
Gender: Male	1.00		1.00	
Female	1.24(1.14–1.34)	<0.001	0.92(0.79–1.06)	0.248
Age: (per year)	1.00(0.99–1.01)	0.960	0.99(0.98–1.00)	0.002
CD4 count: (per 100)	0.69(0.66–0.72)	<0.001	0.74(0.68–0.80)	<0.001
Weight (kg): <45	1.00		1.00	
45–49	0.98(0.88–1,11)	0.793	1.02(0.85–1.24)	0.811
50–59	1.04(0.93–1.15)	0.510	0.88(0.72–1.07)	0.186
≥60	1.27(1.14–1.41)	<0.001	0.81(0.64–1.01)	0.062
Diagnosis of WHO Stage 3 Condition	1.25(1.06–1.47)	0.007	1.21(0.91–1.60)	0.191
Diagnosis of WHO Stage 4 Condition	1.63(1.22–2.17)	0.001	1.70(1.10–2.61)	0.016

## Discussion

Excess mortality during the early months of ART is a key driver of divergent outcomes for people treated with ART in RLS compared with high-income countries [1]. The 1-year mortality in this study was comparable to that previously reported in similar cohorts [1], [4]. Previous studies have demonstrated the importance of pre-treatment CD4 cell count, gender and age as influences of mortality during this critical period [3], [4]. In this large study of adults and adolescents initiating ART in RLS, we have shown that in addition to these factors, a new diagnosis of a WHO stage 3 or 4 condition more than doubled mortality during the first six months of treatment. Mortality was more strongly associated with stage 4 than stage 3 conditions and was associated with most WHO stage 4 conditions, but only two of the WHO stage 3 conditions, namely, pulmonary TB and oral candidiasis. This confirms the overall value of the WHO clinical staging system after treatment initiation.

The diagnosis of an opportunistic infection during early ART is often considered a manifestation of immune reconstitution disease in which the immune effects of HIV suppression result in enhanced immune responses and ‘unmasking’ of previously unrecognized infections [16]. A proportion of these presentations are, however, also likely to be predominantly due to pathogen virulence in the setting of persistent or residual immune deficiency. It is therefore preferable to consider these clinical presentations as ‘ART-associated’ with poorly understood, but likely heterogeneous pathogenesis, driven by high prevalence of infections and advanced HIV disease at time of ART initiation [17], [18].

As in most RLS, screening for opportunistic infections prior to ART commencement in our study was predominantly on the basis of clinical symptoms only. The protean nature of opportunistic infection clinical presentations in the setting of advanced HIV disease and the limited available diagnostic capacity results in missed opportunities for prompt diagnosis and treatment of opportunistic infections prior to ART initiation. A recent study suggests that up to half of the TB diagnosed in the first year of ART may actually be sub-clinical disease diagnosable prior to ART initiation [19]. Enhanced screening for other infections prior to commencement of ART is also likely to lead to earlier diagnosis and treatment, which may reduce the high mortality rates demonstrated in our study and elsewhere [1], [4]. Three-quarters of our study population initiated ART with a CD4 count below 200/μL. Reducing excess mortality during early ART will require concerted efforts, funding and improved healthcare systems to ensure earlier diagnosis of HIV infection and effective linkage to care, as well as ongoing access to ART for individuals not yet receiving treatment.

In this cohort Asian patients had a lower median CD4 count at ART initiation and were more likely to have WHO stage 4 disease than African patients. This is likely to represent poorer access to ART in Myanmar during the study period and treatment programs prioritizing available ART to patients with more advanced disease. There are also a lower proportion of women in the Asian cohort who may be diagnosed earlier than men due to antenatal screening.

Despite patients having a lower median baseline CD4 count at the start of ART in Asian programs, rates of mortality and LFU were lower than in African programs. This could reflect higher underlying mortality if undiagnosed infections were more common in Africa compared with Asia, or reduced access to quality care related to poorer health care infrastructure, fewer qualified human resources, limited access to diagnostic tools, and increased workload due to the higher prevalence of HIV infection in Africa.

Extra-pulmonary cryptococcosis, predominantly cryptococcal meningitis, was the condition most strongly associated with mortality in this study. Fluconazole is often used to treat cryptococcal meningitis in RLS due to its ease of use, relative safety and availability and was often used in the clinics in this study. However outcomes with fluconazole treatment are inferior [20], [21] and recent WHO guidelines [22] recommend combined amphotericin and 5-flucytosine, that are either not registered, available, affordable or feasible in many RLS [21]. Recent data also suggests that fluconazole should be used at a dose of at least 800mg/day [21], [22], which is higher than the dose of 400mg/day generally used in RLS, including in this study. Screening for cryptococcal disease has not been widely available in RLS, but it has been shown that a positive serum cryptococcal antigen test prior to commencement of ART is highly sensitive for predicting the development of cryptococcal meningitis and that screening those with CD4 <100 cells/μL is a cost effective measure to reduce cryptococcal disease [23], [24].

TB has previously been associated with increased mortality during early ART in RLS [7], [25]. It was seen commonly in our study population and was associated with the greatest absolute number of deaths. The WHO clinical staging system designates pulmonary TB as a stage 3 condition and extra-pulmonary TB as a stage 4 condition [26], whereas both forms of TB are designated as category C conditions in the United States Centers for Disease Control HIV classification system [27]. We found that during early ART pulmonary and extra-pulmonary TB had similar impact on mortality which was higher than that for most WHO stage 3 conditions and lower than that for most WHO stage 4 conditions. The mortality risk associated with the condition of extra-pulmonary TB does not, however, adequately reflect the underlying heterogeneity of this condition. In an analysis of 769 patients with TB-HIV co-infection in Thailand, meningeal TB was associated with increased mortality relative to pulmonary TB, but intra-abdominal, disseminated and lymphatic TB were not [28].

Strategies to reduce the burden of TB and TB-associated mortality are important in efforts to reduce mortality during ART. Screening with symptoms and sputum smear microscopy both have a low sensitivity for diagnosis of TB in patients infected with HIV [19]. Two evaluations of WHO clinical algorithms for smear negative TB in HIV positive patients showed adequate negative predictive value but poor positive predictive value [29], [30]. New molecular diagnostics such as the Xpert MTB/Rif system that use real-time polymerase chain reaction technology have been shown to have improved sensitivity for diagnosis of smear negative disease in patients with HIV [31] and this system is now recommended by WHO [32], but is still not widely available.

In this study HIV wasting syndrome was associated with a high mortality risk and is therefore a marker of significantly reduced prognosis after ART initiation and appropriately designated as a stage 4 condition. In settings with limited diagnostic capacity, HIV wasting syndrome is likely to represent a number of difficult to diagnose conditions, including TB which may be drug resistant, MAC, cytomegalovirus infection and HIV itself. Interestingly, in our analysis HIV wasting syndrome had a similar association with mortality as a diagnosis of non-tuberculous mycobacterial infection, which is often suspected to be the cause of this syndrome. The individual components of the HIV wasting syndrome were associated with mortality on univariate analysis only, supporting their designation as stage 3 conditions.

In this analysis WHO stage 3 conditions were not associated with increased mortality on multivariate analysis, except for pulmonary TB and candidiasis. Oral candidiasis has previously been associated with mortality in a small study from South Africa [33]. It is possible that this condition serves as a surrogate marker for the presence of other undiagnosed opportunistic infections and given it is relatively common and easily diagnosed could be used by diverse clinical cadre in primary care settings to identify patients at increased risk for adverse outcomes. Interestingly, unexplained neutropenia, anemia or thrombocytopenia was strongly associated with mortality on univariate analysis but this was of borderline statistical significance on multivariate analysis. This may be because this condition often results from HIV infection itself and thus the association with mortality lost statistical significance due to the adjustment for CD4 count in the multivariate model. Severe anemia at baseline has previously been shown to be associated with increased mortality in patients commencing ART [3], [4], [6].

In our study 7.2% of patients were considered LFU. This was comparable to that reported at 1 year by May et al [4] in Sub-Saharan Africa but lower than reported rates of 12–19% in other studies in RLS [1], [5]. It has previously been reported that up to 58% of patients initially considered to be lost to follow-up may actually have died if detailed tracing is performed. [34] Using these figures the true mortality in our population may be as high as 12%. The overall similarity in factors associated with both LFU and mortality supports the association between these factors and mortality, and reinforces the importance of these factors in understanding the causes of adverse outcomes during early ART. A competing risks analysis performed to assess the impact of LFU on the mortality analysis showed the factors associated with mortality remained largely the same.

In contrast to findings in this study a similar analysis performed on data from a cohort in Europe and North America found that a diagnosis of non-Hodgkins lymphoma and progressive multifocal leukoencephalopathy were most strongly associated with mortality after ART initiation in resource-rich settings [35]. The overall mortality in this study was 3.6% over a median follow-up of 43 months, which was lower than our study and the median CD4 count at initiation of therapy was higher. This is likely to reflect differences in the incidence of endemic pathogens [36] as well as poorer access to both diagnostics and therapeutics in resource limited settings.

This study has several limitations. The diagnosis of HIV associated conditions was at the discretion of treating clinicians, rather than according to pre-specified protocols. MSF has standardized guidelines for diagnosis and treatment of opportunistic infections, but it is likely that variable approaches to diagnosis and treatment were taken. Some conditions such as cytomegalovirus infection are difficult to diagnose and treat in RLS and thus were uncommonly diagnosed. Treatment of conditions would have also varied between sites depending on the availability and ability to administer some therapeutic agents such as amphotericin. Nevertheless we believe the limitations in the provision of treatment at some sites would be representative of difficulties seen in many other RLS. Also low body weight, which has often been associated with mortality in people with HIV [3], [4], was associated with mortality in the univariate analysis, but had to be excluded from the multivariate analysis for statistical reasons. It is possible this was due to a different association between weight and mortality in Asia compared with Africa. The lack of pre-ART data precluded comparison of mortality rates before and after initiation of ART, and an assessment of associations of specific WHO conditions diagnosed pre-ART with mortality on ART. Finally, the Asian data included in our study was derived from programs mainly operating in Myanmar, limiting the generalisability of our findings to other settings in this region, particularly middle-income countries. Data from a program in Moldova was included to increase power for the primary analysis however there were insufficient patients from Moldova to allow a meaningful comparison of Eastern European patients with those from Africa or Asia.

In conclusion this study has demonstrated that patients commencing ART in RLS are exposed to a high risk of mortality in the early ART period and that specific WHO stage 3 and 4 conditions contribute significantly to this risk. Understanding the relative contribution of these conditions to mortality during early ART will assist with initiatives to reduce excess mortality during this period, including prioritization of resources for diagnostics, treatment and research. Strategies to reduce mortality during early ART are needed, including earlier HIV diagnosis and linkage to care, ongoing commitment to ART access and improved screening and treatment of opportunistic infections.
